# Two Cases of Congenital Chylethorax: A Successful Story of Medical Management

**DOI:** 10.1155/2021/6634326

**Published:** 2021-08-07

**Authors:** I. Kankananarachchi, K. K. S. Priyankara, K. K. K. Lakman, K. Withanaarachchi, P. K. G. Gunathilaka

**Affiliations:** ^1^Department of Paediatrics, Faculty of Medicine, University of Ruhuna, Matara, Sri Lanka; ^2^Paediatric Pulmonology Unit, Teaching Hospital Karapitiya, Galle, Sri Lanka; ^3^Neonatal Intensive Care Unit, Teaching Hospital Karapitiya, Galle, Sri Lanka

## Abstract

Congenital chylothorax (CC) is one of the most common causes of pleural effusions in neonates. Associated ipsilateral pulmonary aplasia in CC results in neonatal respiratory distress. Here, we report 2 cases of CC who were managed in the Teaching Hospital Karapitiya, Sri Lanka, between 2017 and 2019. Both babies were males who presented with respiratory distress within a few hours of birth. Their antenatal ultrasound scans failed to detect CC. Chest radiographs showed left-sided pleural effusions. Pleural fluid was milky yellowish suggestive of chylothorax, and the analysis revealed elevated triglycerides, high lymphocyte counts, and low cholesterol levels compatible with CC. They were managed in the neonatal intensive care unit and kept nil by mouth for initial 48 hours. Intravenous octeotride infusion was started on day one and was continued for 7 and 10 days, respectively. The maximum dose of octeotride was 2 *μ*g/kg/hour. Both babies needed intercostal tube placement for 5 and 6 days, respectively. None of them required invasive ventilation. They were started on a medium-chain fatty acid formula, which was continued for about one week. Both babies were commenced on breast milk by day 7 of life and continued with exclusive breastfeeding. Within two weeks, they were discharged home and followed up in the paediatric respiratory clinic for another year. None of them was found to have long-term respiratory complications during the follow-up.

## 1. Introduction

Congenital chylothorax is the most common cause of pleural effusions in newborn babies, in which predominant lymphocytic fluids accumulate in the pleural space. The prevalence of CC is 1 : 10000, and it has a broad spectrum of clinical presentation ranging from asymptomatic to no immune hydrops fetalis [1]. CC can complicate with poor lung development resulting in respiratory distress in the newborn period. Occasionally, CC is associated with other syndromes [2]. Here, we report 2 cases of congenital chylothorax that were managed in the TH Karapitiya, Sri Lanka, between 2017 and 2019.

## 2. Case 1

**Figure 1:**
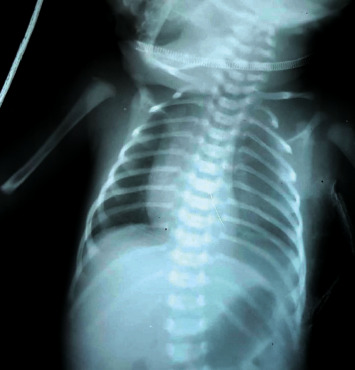
Left-sided pleural effusion of case 1.

A baby boy was born at term to a 30-year-old mother via normal vaginal delivery with a birth weight of 2.9 kg. The antenatal ultrasound scans did not detect any congenital abnormality. He developed respiratory distress soon after birth and was taken to the special care baby unit. On examination, there was reduced air entry over the left-side hemithorax. His chest X-ray (CXR) showed a large left-sided pleural effusion which was confirmed by ultrasonography (Figure 1). He continued to have respiratory distress and required nasal prong oxygen 2 L/min. However, the baby did not need any invasive ventilator support. The pleural fluid was milky, and the analysis showed elevated triglycerides and low cholesterol levels. The cell count, electrolytes, and amylase level in the pleural fluid was within the normal range. His basic serum biochemical and haematological markers were normal. The diagnosis of chylothorax was made based on the radiological and pleural fluid analysis.

He was started on IV octeotride infusion 2mic/kg/min on day one and continued for 48 hours. An intercostal tube was placed on the same day. During the octeotride infusion, there was a significant reduction of the chylous output and marked clinical improvement. However, within 12 hours of discontinuing the octeotride infusion, he developed respiratory distress and increased chylous output. The repeat CXR showed worsening of chylothorax. IV octeotride infusion was restarted at the same dose and continued for a total of 7 days. From the 3rd day onwards, he was started on the medium-chain fatty acid-rich formula and continued for up to 7 days. He was on nasal cannula oxygen for the initial four days, and the support was weaned off gradually. The baby was discharged on day 12, and he was on established breastfeed at that time. The IC tube was removed after five days. At the time of data collection, he was 18 months and had no concerns during the follow-up.

## 3. Case 2

**Figure 2:**
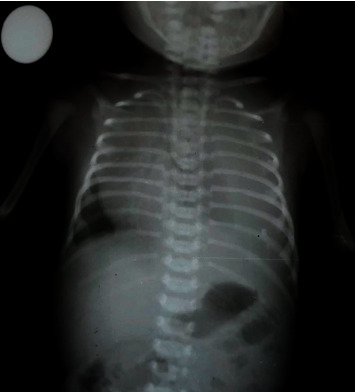
Left-sided pleural effusion of case 2.

A male infant was born via an emergency caesarian section (LSCS) at 38 weeks of gestation with a birth weight of 3.1 kg. The LSCS was performed due to fetal distress, but the baby had an average APGAR score at birth. His mother was a 26-year-old prim gravida who had an uneventful antenatal period. The baby developed respiratory distress soon after birth and was taken to the neonatal intensive care unit. He was started on nasal prong oxygen and then needed high-flow nasal cannula oxygen. His chest X-ray showed a massive left-sided pleural effusion (Figure 2). Echocardiography did not show any underlying heart defects.

The pleural fluid was milky, and the analysis of it revealed elevated triglycerides and low cholesterol levels. The cell count, electrolytes, and amylase level were within the normal range (Table 1). The diagnosis of chylothorax was made, and an IC tube was placed on day 1. He was kept nil by mouth for 48 hours and started on IV octeotride infusion at 2mic/kg/min. On day three, he was commenced on an MCFA-rich formula, and by day seven, he was started on breast milk. The IC tube was removed on day six, and the repeat chest X-ray showed no effusions.

IV octeotride infusion was continued for a total of 10 days, and he was discharged on day 14. On discharge, he had regained birth weight and while on exclusive breastfeeding for a week.

He was eight months at the time of data collection and he had normal growth and development without any evidence of chronic lung disease.

## 4. Analysis of the 2 Cases

Both patients were males, and the abnormality was not detected in the antenatal period. Chylothorax was noted on the left side on both occasions. None of them had structural heart defects, lymphopenia, or hypoalbuminemia (Table 1). Though they were managed in the neonatal intensive care units, none of them required invasive ventilation. The patients responded well to octeotride therapy and the MCFA-rich formula. They went home within two weeks, and none of them had long-term respiratory problems.

## 5. Discussion

CC is a rare presentation in children with mortality between 20%–60% [1]. When it is associated with hydrops fetalis, the mortality is 98% [2]. Pulmonary hypoplasia and congestive heart failure are the main reasons for the increased mortality. In addition, CC can be associated with other genetic syndromes such as trisomy 21, Noonan syndrome, and Turner syndrome [2]. In this case, both patients survived without any long-term complications, and none of them was syndromic. However, in some case series, the mortality and morbidity of the condition were much higher than this [3, 4]. Helen H et al. have published a case series of 5 neonates with CC in 2017 in which all babies had antenatally detected hydrops fetalis [5]. One baby died, and the length of hospital duration ranged from 53–144 days. In contrast, none of these two cases had hydrops fetalis, and the hospital stay went between 10–21 days.

The main aims of the management of CC are to remove already accumulated fluids, prevent recollection, manage associated complications, and look for the underlying aetiology while maintaining optimum nutrition. IC tube placement and continuous suction drainage are the methods to remove already fluids in the space. Occasionally, redirection of chyle using pleuroperitoneal shunt or diaphragmatic fenestration will be useful to manage respiratory distress. A low-fat diet, MCFA-rich formula, and octeotride therapy are widely used to prevent further accumulation of fluids.

The exact mechanism of action of octeotride in the management of CC is not known; however, it is believed that it causes vasoconstriction in the splanchnic vessels by reducing the intestinal blood flow resulting in reduced production of chyle. The use of octeotride for CC was first reported by Young et al. in 2004, and they have used subcutaneous octeotride at a dose of 40mic/kg/day to 70mic/kg/day [6]. Ever since then, octeotride has been used to manage CC both subcutaneously and intravenously with promising outcomes. However, there are no randomized controlled clinical trials to evaluate the medication's efficacy, dose, duration, and safety profile. In most cases, intravenous octeotride has been given as a continuous intravenous infusion between 0.3 and 10 *μ*g/kg/hour [7]. In our case series, the highest rate we used was 2 *μ*g/kg/hour. The duration of therapy is usually based on clinical response. In the literature, octeotride had been continued for 4–21 days. Similarly, in our case, octeotride usage was between 7 and 10 days. Arrhythmias, hyperglycemia, pulmonary hypertension, and necrotizing enterocolitis are known side effects of Octreotide [2]. However, in this case series, except for some recordings of elevated blood sugar levels, other side effects were not reported. Sildenafil and sirolimus have been successfully used in some cases when there is no improvement for octeotride [8, 9].

The maintenance of nutrition is an essential aspect of CC. Intravenous fluids, MCFA-rich formula, and parenteral nutrition are the available treatment modalities.2 MCFA-rich formulas with 8–12 carbon chains can bypass the lymphatic system and are directly absorbed into the portal venous system. In this case series, all patients were started on Pregestimil and converted to breastfeeding within seven days. In the absence of a special formula, fortified skimmed breast milk can be used as an alternative in resource-limited settings [10].

Surgical interventions or chemical pleurodesis are indicated in patients who are not responding to conservative management. Thoracic duct ligation and thoracoscopic pleurodesis are the available surgical options. However, there is no consensus about the exact timing of the surgery [11]. Povidone iodine, picibanil, oxytetracycline, and talc are being used in chemical pleurodesis [2].

## 6. Conclusions

CC can be successfully managed with Octeotride, MCFA formula, and other supportive therapy. In the absence of other associated abnormalities, CC carries a better prognosis.

## Figures and Tables

**Table 1:** Investigation summary of 2 cases.

		Case 1	Case 2
Pleural fluids analysis	Erythrocytes (/*μ*L)	260	340
	White cell count (/*μ*L)	1200	1320
	Neutrophils : lymphocytes	10% : 80%	4% : 90%
	Protein (mg/dL)	35	42
	Cholesterol (mg/dL)	80	90
	Triglycerides (mg/dL)	420	490
	Chloride (mmol/L)	113	110
	Glucose (mg/dL)	78	89
	Lactate dehydrogenase (LDH)	189	232

Serum	LDH (0–248 U/L)	480	560
	Triglyceride (mg/dL)	90	120
	Cholesterol (mg/dL)	115	110
	Albumin (mg/dL)	42	48

## Data Availability

The data used to support the findings of this case report are available upon request from the corresponding author.
